# A Preliminary Study on the “Hitchhiking” of Radionuclides on Microplastics: A New Threat to the Marine Environment from Compound Pollution

**DOI:** 10.3390/toxics13060429

**Published:** 2025-05-24

**Authors:** Chaoran Li, Zhonglai Zhou, Xinran Meng, Junheng Li, Hongyi Chen, Tianle Yu, Min Xu

**Affiliations:** 1 Jiangsu Key Laboratory of Ocean-Land Environmental Change and Ecological Construction, School of Marine Science and Engineering, Nanjing Normal University, Nanjing 210023, China; 242602024@njnu.edu.cn (Z.Z.); x.meng0720181@arts.ac.uk (X.M.); ytlll888@126.com (T.Y.); 2 UK Dementia Research Institute, Department of Brain Sciences, Imperial College London, London SW7 2AZ, UK; junheng.li17@imperial.ac.uk; 3 Department of Computer Science, University College London, London WC1E 6BT, UK; hongyi.chen.16@ucl.ac.uk

**Keywords:** microplastics, radionuclides, compound pollution, marine ecosystems

## Abstract

With the widespread use of plastic products globally, the issue of microplastics as environmental pollutants has become increasingly severe. Due to their small size, large surface area, and hydrophobic properties, microplastics are capable of adsorbing various pollutants, particularly radionuclides, which, in turn, can impact the stability of ecosystems. This laboratory study investigates the adsorption capacity of microplastics (PVC) for radionuclides (Ra-226, Cs-137, and K-40) under controlled conditions, examining the effects of spatial distribution and particle size. The laboratory experiment results indicate that the adsorption of Ra-226 by microplastics was significantly higher in the bottom water compared to the surface layer, with concentrations of 13.29 mBq/kg on microplastics mixed with the bottom water and 1.65 mBq/kg in the surface layer. The concentration of Cs-137 on microplastics mixed with the bottom water was 6.99 mBq/kg, while on microplastics mixed with the surface water, the concentration was 1.31 mBq/kg. In contrast, the adsorption of K-40 was lower, with concentrations of 2.1 mBq/kg and 0.35 mBq/kg on microplastics mixed with the bottom and surface water, respectively. Furthermore, microplastics with smaller particle sizes exhibited stronger adsorption capacities. The adsorption concentrations of Ra-226 and Cs-137 by 50 µm microplastics were 13.29 mBq/kg and 6.99 mBq/kg, respectively, while the concentrations for 100 µm and 150 µm particles decreased to 3.14 mBq/kg and 1.39 mBq/kg, and 2.2 mBq/kg and 0.35 mBq/kg, respectively. These findings suggest that the adsorption capacity of microplastics is significantly influenced by particle size and sediment depth, highlighting the potential risk of exacerbating the spread of radioactive pollutants in marine ecosystems.

## 1. Introduction

As the demand for plastic products grows, plastic production has also increased. Due to its corrosion resistance, low conductivity, and low cost, plastic is widely used in daily life. However, the low recycling rate and high costs associated with plastic recycling result in inefficiencies in plastic waste management. According to current research, approximately 14 million tonnes (Mt) of plastic waste enters the ocean annually, leading to the continuous accumulation of marine plastic waste [1,2]. Factors such as ultraviolet radiation, sea waves, and biological activity contribute to the fragmentation of plastic into smaller microplastics, which can eventually degrade into nanoscale plastics [3,4]. Due to their slow degradation, microplastics persist in the environment for extended periods and are eventually ingested by marine organisms, posing a threat to marine biodiversity and human health [5,6].

Radionuclides originate from both natural and anthropogenic sources, referring to unstable elements that release alpha (α), beta (β), and gamma (γ) radiation during their decay process. Naturally occurring radionuclides, such as uranium-238 (U-238) and radium-226 (Ra-226), primarily emit alpha radiation, while anthropogenic radionuclides like cesium-137 (Cs-137) and strontium-90 (Sr-90) predominantly emit beta radiation. Additionally, many radionuclides, including potassium-40 (K-40) and cobalt-60 (Co-60), emit gamma radiation, which has strong penetrating power and is commonly used in medical and industrial applications [7]. Natural radionuclides exist in the Earth’s crust, with long half-lives and minimal quantities [8]. Anthropogenic radionuclides are primarily released through industrial activities, such as nuclear facility emissions, and they eventually enter the ocean, accumulating in marine sediments [9,10]. These radionuclides can transfer through the food chain, accumulating in marine organisms and potentially causing toxic effects such as DNA damage, oxidative stress, and metabolic disruption. Their prolonged presence in marine environments can alter ecosystem functioning, reduce biodiversity, and negatively impact marine food webs, ultimately posing long-term risks to both marine life and human health [11].

The Yellow Sea is a typical semi-enclosed marginal sea in the western Pacific, bordered by the Chinese mainland and the Korean Peninsula [12,13]. This region is economically developed with frequent industrial activities, leading to significant impacts on the marine ecosystem [13,14]. The waters around Haizhou Bay, Lianyungang, are of particular research interest due to their abundant resources and critical infrastructure, such as the Tianwan Nuclear Power Plant. Studies have shown that microplastic pollution in Lianyungang is higher than in most marine areas of China and other countries [15,16]. Among these areas, the seawater aquaculture zones and artificial reef zones exhibit particularly severe pollution, indicating the significant impact of marine aquaculture on microplastic contamination [17].

There has also been growing attention paid to the study of radionuclides in the waters surrounding the Tianwan Nuclear Power Plant. Yuyue et al. assessed the impact of radioactive Sr-90 and Cs-137 in these waters through monitoring data, suggesting that long-term low-level emissions pose minimal ecological risks [18]. Although the concentration of radionuclides is relatively low and does not present significant health risks to humans, it provides a foundation for further assessing the compound hazards of microplastics and radionuclides [19,20].

Microplastics, due to their small size, high porosity, large surface area, and hydrophobic nature, can adsorb various pollutants, including radionuclides, which have been detected in microplastic samples collected from marine environments [21,22]. Previous studies have focused primarily on the adsorption of organic pollutants by microplastics, with less attention given to metal pollutants and radionuclides [23]. In 2010, Ashton first reported the adsorption of heavy metals onto plastics [24]. In recent years, research on the interactions between microplastics and radionuclides has gradually increased [25], revealing that microplastics can significantly adsorb radionuclides, with the adsorption mechanism being influenced by the affinity between metals and the plastic surface [24,26]. The concentration of heavy metals generally decreases with distance from the coastline, as heavy metals tend to settle due to their higher density, with chemical precipitation—such as the formation of metal hydroxides, sulfides, and carbonates—also playing a crucial role in their accumulation in sediments [27]. Qiao et al., through simulations of radionuclide transport in the ocean, found that their migration is slow, and they rapidly adsorb onto suspended particles [28,29]. Otosaka et al. studied radioactive cesium in sediments near the Fukushima Nuclear Power Plant and found that the total amount of cesium was only 2% of the accident’s total emissions [30,31]. The adsorption and migration process of microplastics and radionuclides provides a theoretical basis for environmental risk assessments [32].

Different types of microplastics exhibit varying adsorption capacities for radionuclides. For example, microplastics such as polyethylene (PE) and polyvinyl chloride (PVC) can serve as carriers for radionuclides like U-232 and Cs-137 [33,34]. Ioannidis et al. found through experiments that the adsorption capacity of microplastics for radionuclides varies under different pH conditions, showing an increasing trend with rising pH, reaching its peak at pH 9 [33,35]. Research has also shown that modifying the surface of microplastics significantly enhances their adsorption capacity for radionuclides [35].

Overall, the combination of microplastics and radionuclides may pose a severe threat to marine environments. The synergistic effect of microplastics, especially their adsorption of radionuclides, could potentially pose risks to human health through the food chain [36,37]. The microplastic pollution in Haizhou Bay, Lianyungang, and the waters around the Tianwan Nuclear Power Plant is significant, with marine aquaculture activities playing a major role in the contamination. Despite the relatively low concentrations of radionuclides, environmental studies have shown that microplastics in marine waters can become contaminated with these radioactive elements, raising concerns about their long-term ecological and health risks.

Currently, research on the interactions between microplastics and radionuclides is limited, with most studies conducted under controlled laboratory conditions that fail to capture the complexity of real marine water. Additionally, studies in the Yellow Sea, especially in the waters surrounding the Tianwan Nuclear Power Plant, are particularly scarce. This study provides novel insights by conducting field-based investigations, offering empirical data on the adsorption characteristics of radionuclides (e.g., Cs-137, Ra-226, and K-40) onto microplastics (especially PVC) in real seawater conditions. The findings contribute to understanding the compound risks posed by microplastics and radionuclides in a heavily industrialized marine region.

## 2. Method

### 2.1. Sample Preparation

**Figure 1:**
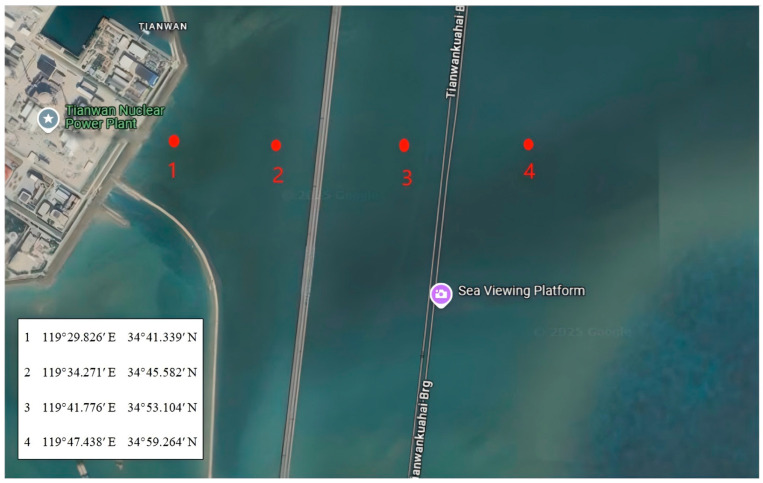
Schematic diagram showing the locations of the four sampling points around the Tianwan Nuclear Power Plant.

**Figure 2:**
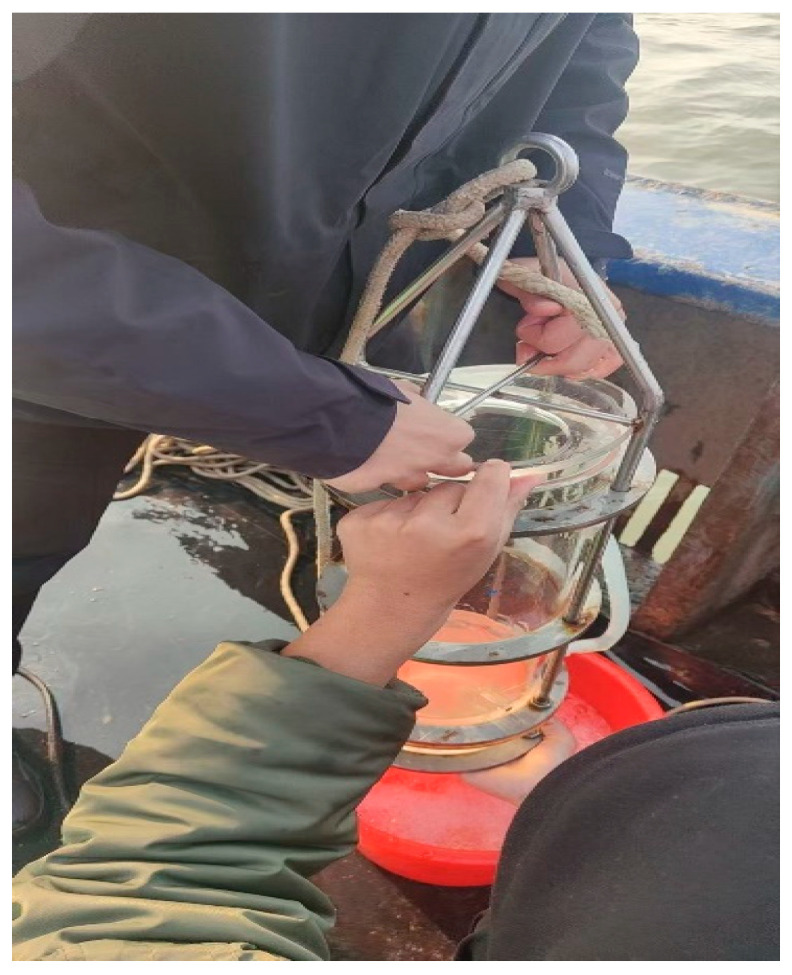
On-site collection of experimental samples using a glass water sampler.

The microplastic (PVC) samples were obtained from Xutai Microplastic Co. (Dongguan, China), with particle sizes of 50 µm, 100 µm, and 150 µm. PVC, a high-density polymer, was chosen due to its widespread use, environmental persistence, and strong adsorption potential for radionuclides. Prior to the experiment, all microplastic samples were cleaned using deionized water and an ultrasonic cleaner to remove surface impurities. The water samples were collected from four sampling points around the Tianwan Nuclear Power Plant (Lianyungang, China) (Figure 1) using a glass water sampler (Denuo Co., Xinxiang, China), which could be opened and closed at a specific depth (Figure 2). At each point, 10 L of water was collected from both the surface and bottom layers, with depth variations depending on the location. At sampling point 4 (depth: 23 m), 10 L of water was collected from the surface and the bottom layer at 20 m. At sampling point 3 (depth: 13 m), 10 L was collected from the surface and the bottom layer at 12 m. At sampling point 2 (depth: 6 m), 10 L was taken from both the surface and the bottom layer at 6 m. At sampling point 1 (depth: 4 m), 10 L of water was collected from the surface and the bottom layer at 4 m (Table 1). Immediately after collection, the water samples were filtered using a 20 cm, 800-mesh stainless steel sieve to remove large particulate impurities and stored in clean glass containers. The collected natural water samples were stored at −4 °C for 24 h before further analysis.

The microplastics (25 g) were mixed with 1 L of concentrated seawater (at a 1:10 concentration ratio) and stirred mechanically for 2 h at a room temperature of 25 °C, pH 8.1, salinity 35 PSU to ensure adsorption equilibrium [33]. This concentration process was based on preliminary experiments, considering the radionuclide levels in the seawater near the Tianwan Nuclear Power Plant (Ra-226 ~5.68 mBq/L, Cs-137 ~1.79 mBq/L, K-40 ~1.11 mBq/L). A blank control was conducted using ultrapure water under the same conditions with the same microplastic samples, and the final measured adsorption values were background-corrected by subtracting the radionuclide levels detected in the blank samples. Each treatment involved three sets of parallel experiments to ensure data reliability and reproducibility. The microplastics were then filtered, dried, and stored at −4 °C for further analysis.

### 2.2. Instrument Setup and Measurement

All microplastic samples were analyzed for gamma radiation using a GR4020 High-Purity Germanium γ-spectrometer (Canberra, Meriden, CT, USA). The instrument setup was as follows: relative efficiency of 40%, energy resolution @122 keV of 1.0 keV, peak shape @122 keV of 2.0 keV, and peak-to-Compton ratio of 56. The measurement time for each sample was set between 300 and 1800 s, depending on the activity levels of the detected radionuclides. Lower-activity samples required longer counting times to achieve an adequate signal-to-noise ratio and ensure reliable quantification, whereas higher-activity samples could be accurately measured within a shorter duration. This adaptive measurement approach minimized statistical uncertainties while optimizing efficiency in data acquisition.

Samples were placed in front of the detector window, maintaining a distance of 5–10 cm to ensure optimal signal collection. The measurement was performed within an energy window of 100–1500 keV to capture characteristic gamma-ray peaks for radionuclides such as Ra-226 (186.2 keV, 226.1 keV), Cs-137 (661.6 keV), and K-40 (1460 keV). Background spectra were collected before sample measurements and subtracted from the final data to eliminate environmental interference.

### 2.3. Data Analysis

The obtained gamma spectra were processed using specialized spectrum analysis software (Canberra Spectrum Analyzer, Genie™ 2000 4.0) to identify the characteristic peaks of Ra-226, Cs-137, and K-40, and the area of each peak was calculated using a Gaussian fitting method. The radionuclide concentrations in each sample (in units of mBq/kg) were then calculated based on the known activity of standard sources. Data were analyzed using the standard deviation and relative standard deviation (RSD) to assess the repeatability and accuracy of the experiments.

To examine the differences in adsorption capacities among microplastics of different particle sizes, independent experiments were conducted for microplastics with particle sizes of 50 µm, 100 µm, and 150 µm (PVC). ANOVA (analysis of variance) was used to assess the significance of the adsorption capacity differences between the different particle sizes at a significance level of *p* < 0.05. To further investigate the differences between specific groups, Tukey’s HSD test was conducted.

### 2.4. Adsorption Isotherms Method

To evaluate the adsorption behavior of radionuclides onto microplastics (PVC) under controlled aquatic conditions, batch adsorption experiments were carried out using Ra-226, Cs-137, and K-40 as target isotopes. The experiments were conducted at a constant room temperature of 25 °C, with a pH of 8.1 and a salinity maintained at 35 PSU, simulating a typical marine environment.

Water samples were collected from four distinct sampling locations, with each point characterized by a different initial radionuclide concentration (*C*_0_), as follows:

- Point 1: Ra-226 = 25.72 mBq/L, Cs-137 = 11.53 mBq/L, K-40 = 6.19 mBq/L;
- Point 2: Ra-226 = 9.51 mBq/L, Cs-137 = 5.26 mBq/L, K-40 = 3.11 mBq/L;
- Point 3: Ra-226 = 6.14 mBq/L, Cs-137 = 2.59 mBq/L, K-40 = 1.28 mBq/L;
- Point 4: Ra-226 = 2.89 mBq/L, Cs-137 = 1.35 mBq/L, K-40 = 0.56 mBq/L.

Each sample was treated with microplastics of three size ranges: 550 µm, 100 µm, and 150 µm, composed of polyethylene spheres. The microplastics (25 g) were mixed with 1 L of concentrated seawater (at a 1:10 concentration ratio) from each point. The mixtures were sealed and agitated in a thermostatic shaker for 24 h to allow for adsorption equilibrium.

After the contact period, the suspensions were filtered using 0.45 µm membranes. The residual radionuclide concentration in the filtrate (*C_e_*) was measured using gamma spectroscopy. The amount of radionuclide adsorbed onto the microplastics (*q_e_*) was calculated according to the following:

(1) $$q_{e}=\frac{(C_{0}-C_{e})\cdot V}{m}$$

This formula was derived by Sabyasachi Rout during his investigation into the adsorption capacity of radioactive iodine by two types of microplastics—high-density polyethylene (HDPE) and low-density polyethylene (LDPE)—where he modified the original adsorption coefficient (Kd) model [38]. His research demonstrated that marine exposure of microplastics significantly enhanced their adsorption capacity for iodide ions.

In the above:

- $C_{0}$ = initial concentration (mBq/L),
- $C_{e}$ = equilibrium concentration (mBq/L),
- V = volume of solution (L),
- m = mass of MPs (kg).

All measurements were repeated in triplicate, and the results were averaged with standard deviations.

To construct adsorption isotherms, *C_e_* values were plotted against *q_e_* values for each particle size group. This allowed for a comparison of the adsorption performance across the microplastic sizes. The data were then modeled using both the Freundlich and Langmuir isotherms to explore potential adsorption mechanisms.

## 3. Results and Discussion

### 3.1. Proximity to Nuclear Power Plant and Nuclide Binding

**Figure 3:**
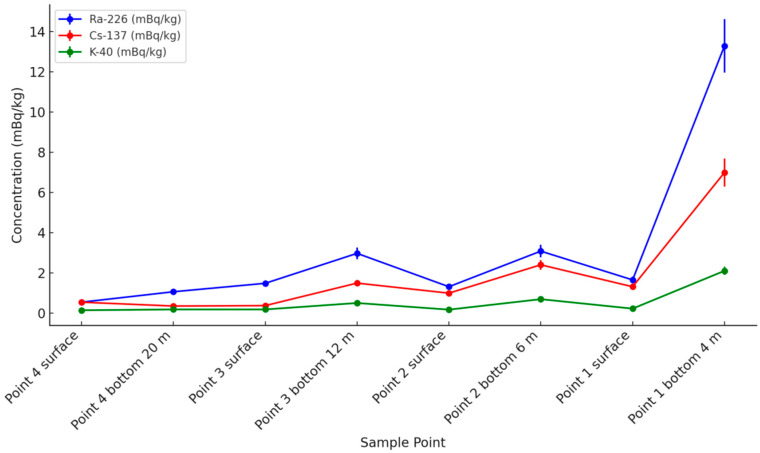
Impact of distance from nuclear power plant on marine microplastic nuclide binding.

As shown in Figure 3, the concentration of Ra-226 was highest at the bottom of sampling point 1, the closest to the nuclear power plant, reaching 13.29 mBq/kg. This concentration is significantly higher compared to the other sampling points (*p* = 0.00543 < 0.01 for point 4 surface, *p* = 0.00558 < 0.01 for point 4 bottom, *p* = 0.00617 < 0.01 for point 3 surface, *p* = 0.00835 < 0.01 for point 3 bottom, and *p* = 0.00584 < 0.01 for point 2 surface), indicating that proximity to the nuclear power plant increased the availability of Ra-226 in the surrounding environment, which, in turn, enhanced its adsorption onto microplastics due to the increased radionuclide concentration and particle interactions in high-exposure areas. However, other environmental factors such as ocean currents, sediment resuspension, and additional contamination sources may also influence the observed distribution and should be considered in further investigations. This phenomenon is consistent with the findings of Jennifer et al. (2015) [39] near the Fukushima Daiichi Nuclear Power Plant. Jennifer et al. observed that within a 1.6 km range from the nuclear plant’s shoreline, the concentration of Cs-137 reached 198 ± 4 Bq/m^3^, which was substantially higher than pre-accident levels, and it gradually decreased with increasing distance from the plant [39]. Similarly, the concentration trend of Cs-137 followed a similar pattern to Ra-226, with the concentration on microplastics mixed with the bottom water at sampling point 1 being 6.99 mBq/kg, displaying a comparable spatial distribution (*p* = 0.00413 < 0.01 for point 4 surface, *p* = 0.00404 < 0.01 for point 4 bottom, *p* = 0.00397 < 0.01 for point 3 surface, *p* = 0.00569 < 0.01 for point 3 bottom, and *p* = 0.00367 < 0.01 for point 2 surface). Cs-137 is commonly found in radioactive wastewater from nuclear power plants. The adsorption of Cs-137 onto microplastics is influenced by factors such as electrostatic interactions and surface functional groups, which facilitate its binding and make microplastics significant carriers for this pollutant. Research by Garraffo et al. in the western Pacific further supports this, suggesting that Cs-137 concentrations decrease as the distance from the nuclear power plant increases [40].

In contrast to Ra-226 and Cs-137, the concentration of K-40 showed relatively stable fluctuations. As a naturally occurring radioactive isotope, K-40 exhibited minimal concentration variation (*p* > 0.05 in all comparisons), which may be attributed to its natural presence in the environment.

Similar phenomena have been observed in studies conducted in other regions. For instance, Nielsen analyzed the distribution of radionuclides in the English Channel and other seas using a box model and found that the concentrations of Cs-137 and Tc-99 were higher in areas closer to the source and gradually diminished with increasing distance [41]. Moreover, Marcus’s study near the UK’s La Hague Nuclear Reprocessing Plant also demonstrated that the concentration of radionuclides decreased with increasing distance from the nuclear source [42]. Research conducted near the Haiyang Nuclear Power Plant in China showed that after an accident, radionuclides spread northeastward along the coastline, with the effects of the flow field and radionuclide decay gradually diminishing over time [29].

### 3.2. Surface vs. Bottom Layer Nuclide Binding

This study involved collecting surface and bottom water samples, then introducing PVC microplastics to examine radionuclide adsorption. As shown in Figure 3, with increasing distance from the nuclear power plant, the concentration of radionuclides in the bottom layer showed an upward trend, particularly for Ra-226 and Cs-137. Statistical analysis (Student’s *t*-test) indicates that the observed differences in Ra-226 (*p* = 0.2717), Cs-137 (*p* = 0.2501), and K-40 (*p* = 0.1785) were not statistically significant, suggesting that additional factors, such as localized depositional processes, may also play a role.

Specifically, the concentration of Ra-226 on microplastics at the bottom of point 1 was 13.29 mBq/kg, while at the surface, it was 1.65 mBq/kg, with the bottom concentration being significantly higher than the surface. This difference indicates that near the nuclear power plant, bottom microplastics had a more pronounced adsorption of Ra-226, likely influenced by the sinking of radionuclide-laden particles, water stratification, and sediment interactions, which contribute to the concentration of radioactive substances in the bottom waters. However, the lack of statistical significance (*p* = 0.2717) suggests that while a trend was observed, additional sampling or analysis is needed to confirm the extent of this effect. Moreover, at other sampling points, the concentration of Ra-226 on microplastics in the bottom water was also generally higher than in the surface layer, consistent with the findings of Wen Yu et al. in the northwest Pacific. They found notable differences in the distribution of radionuclides such as Cs-137 between surface and bottom layers, particularly with increased concentrations in deeper waters [43].

Similarly, Cs-137 concentrations were higher on microplastics mixed with the bottom water than on microplastics mixed with the surface water. At the bottom of point 1, the concentration of Cs-137 was 6.99 mBq/kg, while at the surface, it was 1.31 mBq/kg. This trend reflects the vertical distribution pattern of radioactive substances in seawater, which could be influenced by processes such as particle settling, ocean currents, and water column mixing. Although the concentrations of Cs-137 in the bottom waters appeared higher, statistical analysis (*p* = 0.2501) suggests that the variation between layers may not be significant, indicating the need for further investigation into additional factors affecting radionuclide distribution. These factors may enhance the likelihood of bottom microplastics encountering and adsorbing radionuclides that have accumulated in deeper waters. This phenomenon is in agreement with Aoyama et al.’s study, which found maximum concentrations of radionuclides in subsurface layers at certain depths (e.g., 200–300 m) in the North Pacific, likely associated with the sinking and formation of water masses [44].

In contrast, the concentration of K-40 showed relatively stable fluctuations, with less significant variation between the surface and bottom layers compared to Ra-226 and Cs-137. This was likely due to its natural equilibrium in seawater and its weaker affinity for adsorption onto microplastic surfaces. This suggests that, as a naturally occurring radioactive isotope, K-40 is more evenly distributed in seawater. Unlike Ra-226 and Cs-137, which readily bind to microplastics due to their chemical properties and interactions with plastic surfaces, K-40 exists primarily in ionic form (K^+^) and remains more soluble, reducing its adsorption efficiency on microplastics.

### 3.3. Effect of Different Nuclides on Microplastic Binding

As shown in Figure 3, the concentrations of Ra-226 and Cs-137 at most sampling points were significantly higher than that of K-40, indicating that Ra-226 and Cs-137 have a stronger affinity for microplastics, likely due to their chemical properties, including electrostatic interactions and complexation with functional groups on microplastic surfaces, whereas the binding capacity of K-40 is relatively weaker due to its high solubility and ionic nature. In studies on the adsorption of radioactive substances by microplastics, El Zrelli et al. investigated the interaction between polyethylene terephthalate (PET) plastic bottles and radionuclides and found that the adsorption capacity varied significantly for different radionuclides [37].

Across the different sampling points, the highest concentrations of Ra-226 and Cs-137 were observed on microplastics mixed with the bottom water of point 1, closest to the nuclear power plant, where Ra-226 reached 13.29 mBq/kg and Cs-137 reached 6.99 mBq/kg, while K-40 remained lower at 2.1 mBq/kg. The concentration of these radionuclides generally decreased with increasing distance from the source, highlighting the influence of spatial distribution on radionuclide variability. This result suggests that Ra-226 and Cs-137 interact more readily with microplastics due to their specific adsorption mechanisms, such as surface charge attraction and particle affinity, whereas K-40 remains more dissolved in seawater, reducing its adsorption efficiency. Analysis using the standard deviation and relative standard deviation (RSD) showed that the concentration variability of Ra-226 and Cs-137 differed across the sampling points. The highest variations were observed on microplastics mixed with the bottom water of point 1 (4 m depth), where the standard deviation of Ra-226 was 0.443 mBq/kg and the RSD was 3.34% < 5%. Beyond point 1, the radionuclide concentrations exhibited a general decreasing trend, suggesting that distance from the nuclear power plant and oceanographic factors contribute to their spatial distribution. These data suggest a stronger adsorption capacity of Ra-226 and Cs-137; however, statistical analysis (ANOVA, *p* = 0.1811) indicates that the differences in adsorption among the three radionuclides were not statistically significant, suggesting that additional factors may influence their binding behavior. Wumen’s studies in the Yellow Sea and East China Sea indicated that radionuclides such as Ra-226 and Cs-137 typically have higher concentrations in marine sediments [45,46].

In the comparison between the bottom and surface layers, the Ra-226 concentration on microplastics mixed with the bottom water of point 1 was 13.29 mBq/kg, while on microplastics mixed with the surface water, it was only 1.65 mBq/kg, with a significant difference and an RSD of 5.88%. This difference is related to the deposition process of radioactive substances in the ocean, with more radioactive substances accumulating in the bottom waters, leading to higher concentrations of radionuclides adsorbed by microplastics mixed with the bottom water. Zhao et al. observed a similar deposition process in the East China Sea, indicating that bottom sediments and microplastics have a stronger adsorption effect on radionuclides [47].

The standard deviation was 0.324 mBq/kg, and the RSD was 4.64%, indicating a relatively consistent concentration variation on microplastics mixed with the bottom water. Yuichiro Kumamoto’s research showed that the concentration of Cs-137 is typically higher in deeper waters, particularly in areas impacted by nuclear accidents [48].

The concentration of K-40 fluctuated less between the sampling points and remained lower overall, with no clear spatial trend. This uniformity is likely due to its ionic nature (K^+^), which limits its interaction with microplastic surfaces, in contrast to Ra-226 and Cs-137, whose concentrations were more influenced by proximity to the nuclear power plant and environmental transport mechanisms. However, the ANOVA results (*p* = 0.1811) suggest that the observed differences in adsorption capacities among the radionuclides were not statistically significant, indicating that further investigation is needed. At the bottom of point 1, the K-40 concentration was 2.1 mBq/kg, which was significantly lower than Ra-226 and Cs-137. The standard deviation was 0.092 mBq/kg, and the RSD was 4.38% < 5%, suggesting that the concentration distribution of K-40 is relatively uniform. Ajay’s research in Mumbai Harbor found that the concentration of K-40 is more evenly distributed, which may be one of the reasons for its weaker binding capacity with microplastics [49].

### 3.4. Nuclide Binding on Microplastics of Different Sizes

**Figure 4:**
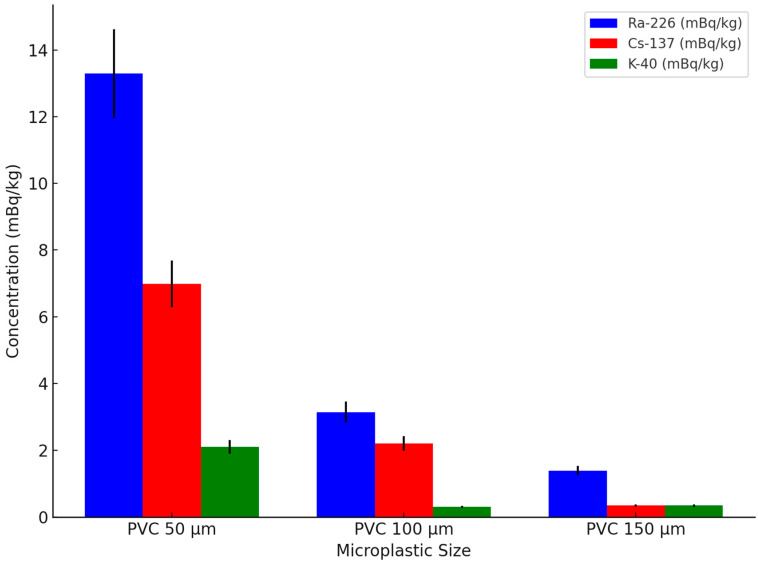
Effect of microplastic size on nuclide binding at point 1 bottom 4 m.

As shown in Figure 4, with an increase in the microplastic particle size, the concentration of Ra-226 and Cs-137 significantly decreased. For example, the concentration of Ra-226 for 50 µm microplastics was 13.29 mBq/kg, while the concentrations for 100 µm and 150 µm microplastics decreased to 3.14 mBq/kg and 1.39 mBq/kg, respectively. This indicates that smaller microplastics more effectively adsorb radionuclides due to their larger surface area, which may increase their potential toxicity when ingested by marine organisms, leading to bioaccumulation and possible disruptions in aquatic ecosystems [50]. According to the results of the analysis of variance (ANOVA), the difference in the adsorption of Ra-226 among microplastic samples of different sizes was significant (*p*-value = 4.06 × 10^−7^ < 0.05), further confirming the effect of particle size on adsorption capacity. Post hoc analysis using Tukey’s HSD test revealed that adsorption on PVC 50 µm was significantly higher than on PVC 100 µm and PVC 150 µm (*p* < 0.05), while the difference between PVC 100 µm and PVC 150 µm was not statistically significant (*p* = 0.0896 > 0.05).

For the concentration of Cs-137, the adsorption concentration for 50 µm microplastics was 6.99 mBq/kg, while for 100 µm and 150 µm microplastics, the concentrations were 2.2 mBq/kg and 0.35 mBq/kg, respectively. This further confirms that microplastics with smaller particle sizes have a stronger affinity for radioactive substances [51,52]. The ANOVA results also show that the difference in Cs-137 adsorption among microplastics of different sizes was significant (*p*-value = 2.08 × 10^−7^ < 0.05). Post hoc analysis using Tukey’s HSD test indicated that Cs-137 adsorption on PVC 50 µm was significantly higher than on both PVC 100 µm and PVC 150 µm (*p* < 0.05). Additionally, adsorption on PVC 100 µm was significantly higher than on PVC 150 µm (*p* = 0.0074 < 0.05). Johansen et al.’s research also indicates that the adsorption capacity of radioactive Cs on PE microplastics coated with a biological film is stronger, with a Kd value as high as 80.3 L/kg, suggesting that biofilm-coated microplastics could enhance radionuclide retention and further increase the risk of radionuclide transfer through marine food webs. Furthermore, under real environmental conditions, factors such as biofilm formation, variations in pH, and salinity fluctuations could influence the adsorption properties of microplastics, potentially altering their radionuclide binding capacity [34].

The concentration of K-40 exhibited less variation, with concentrations of 2.1 mBq/kg, 0.31 mBq/kg, and 0.35 mBq/kg for microplastics of 50 µm, 100 µm, and 150 µm, respectively. However, overall, the adsorption capacity for K-40 was relatively weak, and the differences between particle sizes were small, indicating that while K-40 may pose a lower adsorption risk, Ra-226 and Cs-137 on microplastics could have significant ecological and toxicological implications for marine life.

Ra-226, on the other hand, exhibited much higher concentrations than the other two radionuclides, but its adsorption behavior in natural environments may be further influenced by biological activity, competing ions, and organic matter interactions. Its stronger radioactivity and chemical affinity contribute to its more prominent binding ability on microplastic surfaces [53]. Ra-226 undergoes alpha decay, releasing positive particles, and this charged characteristic may enhance its interaction with the microplastic surface, particularly the negatively charged PVC surfaces. PVC surfaces typically carry a negative charge [50], and negatively charged surfaces are more likely to adsorb particles with stronger charges, especially positively charged alpha particles [53].

### 3.5. Adsorption Isotherms Analysis

This study investigated the adsorption behaviors of Ra-226, Cs-137, and K-40 onto microplastics with different particle sizes (50 µm, 100 µm, and 150 µm), with adsorption data fitted using both the Langmuir and Freundlich isotherms.

**Figure 5:**
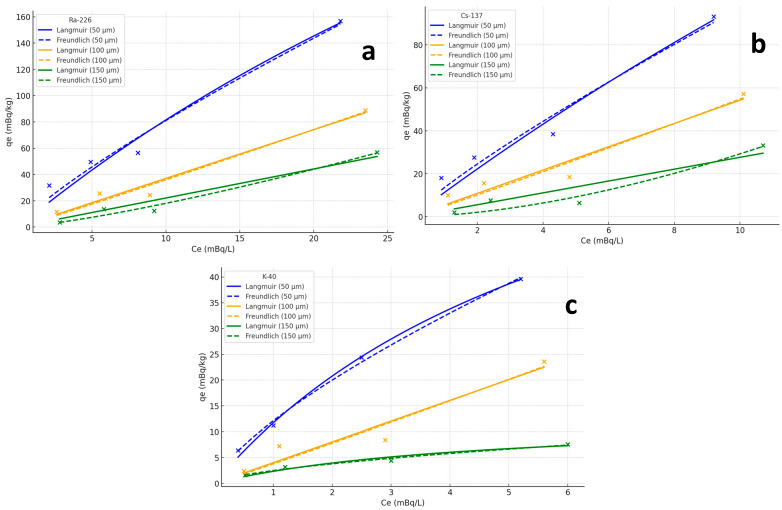
Adsorption isotherms of Ra-226 (**a**), Cs-137 (**b**), and K-40 (**c**) onto polyethylene microplastics of three particle sizes (50 μm, 100 μm, and 150 μm), based on data collected from four sampling points with varying initial radionuclide concentrations (C_0_). Adsorption was conducted at 25 °C, pH 8.1, and salinity 35 PSU. Initial concentrations for each isotope were as follows: Ra-226 (2.89–25.72 mBq/L), Cs-137 (1.35–11.53 mBq/L), and K-40 (0.56–6.19 mBq/L) (see Section 2.4).

The results clearly demonstrate that the adsorption capacities of all three radionuclides decreased with increasing microplastic particle size. For instance, as shown in Figure 5a and Table 2, the adsorption capacity of Ra-226 was significantly higher on 50 µm microplastics (156.8 mBq/kg) compared to 100 µm (88.8 mBq/kg) and 150 µm particles (56.8 mBq/kg) at an initial concentration of 25.72 mBq/L. A similar trend was observed for Cs-137, as shown in Figure 5b and Table 2, where 50 µm microplastics showed the highest adsorption capacity (93.2 mBq/kg), followed by 100 µm (57.2 mBq/kg) and 150 µm particles (33.2 mBq/kg). While K-40, as shown in Figure 5c and Table 2, exhibited a lower adsorption capacity compared to Ra-226 and Cs-137, the pattern of decreasing adsorption with increasing particle size was consistent across all three radionuclides.

The adsorption data were modeled using the Langmuir and Freundlich isotherms. The Langmuir model, describing monolayer adsorption onto a homogeneous surface, follows the following equation:

(2) $$q_{e}=\frac{q_{\max}\cdot K_{L}\cdot C_{e}}{1+K_{L}\cdot C_{e}}$$

The Langmuir model was established by Irving Langmuir in 1916. The core assumption of this model is that adsorption occurs only within a monolayer, and each active site on the adsorbent (i.e., the solid surface) can accommodate only one adsorbate (i.e., a single molecule) [54]. Luke A. Holmes and V. Godoy et al. have employed the Langmuir model to investigate the adsorption of trace heavy metals by microplastics [52,55].

The Freundlich model, which is suitable for heterogeneous surface adsorption, is expressed as follows:

(3) $$q_{e}=K_{F}\cdot C_{e}^{1/n}$$

The Freundlich model was proposed by Hermann Freundlich in 1906. It is an empirical adsorption model based on the assumption that the adsorption process occurs on the heterogeneous surface of the adsorbent and is applicable to both monolayer and multilayer adsorption [54]. In his study, V. Godoy also explored the adsorption of heavy metals by microplastics using the Freundlich model [52].

The fitting results indicate that the Langmuir model provided a better fit for Ra-226 and Cs-137, with the following fitting coefficients:

- Ra-226: $q_{\max}=1275.75$ mBq/kg, $K_{L}=0.00993$ L/mBq;
- Cs-137: $q_{\max}=778.08$ mBq/kg, $K_{L}=0.02185$ L/mBq;
- K-40: $q_{\max}=75.67$ mBq/kg, $K_{L}=0.12457$ L/mBq.

For the Freundlich model, the parameters were as follows:

- Ra-226: $K_{F}=14.84$, 1/n=0.883;
- Cs-137: $K_{F}=18.86$, 1/n=0.867;
- K-40: $K_{F}=8.93$, 1/n=0.724.

The Langmuir model demonstrated a superior fit to the experimental data for Ra-226 and Cs-137, as reflected in higher R^2^ values (R^2^ = 0.982 for Ra-226 and R^2^ = 0.977 for Cs-137). For K-40, the adsorption was comparatively weaker, and the Langmuir model yielded an R^2^ value of 0.965, while the Freundlich model produced an R^2^ value of 0.907, indicating that the adsorption behavior of K-40 is less consistent and more influenced by environmental conditions.

These results underline the significant role of microplastic particle size in determining the adsorption capacity of radionuclides, with smaller microplastics exhibiting enhanced adsorption due to their larger surface area. This has important implications for the potential transport and accumulation of radioactive pollutants in marine environments. The data presented in Figure 5a–c provide a quantitative basis for understanding how particle size influences radionuclide binding, offering insights into the broader environmental risks associated with microplastic contamination.

The adsorption trends captured in this study contribute to a deeper understanding of microplastic–radionuclide interactions in marine ecosystems, highlighting the need for further research to explore the effects of additional factors such as pH, salinity, and the presence of other contaminants. Future studies should also examine the impact of microplastic aging, biofilm formation, and other environmental variables on the adsorption behavior of radionuclides, thus enhancing predictive models for their environmental fate and potential risks to marine organisms.

### 3.6. Toxicological Modeling of Radionuclide-Contaminated Microplastics

To assess the potential biological risks posed by radionuclide-contaminated microplastics, a toxicological exposure model was developed using the experimental adsorption data from 50 µm PVC microplastics. This model estimates the internal radiation dose that a marine organism might receive following chronic ingestion of microplastics containing radionuclides such as Ra-226, Cs-137, and K-40. The estimated dose was calculated based on a simplified ingestion-exposure scenario, assuming a daily intake of 0.5 g of microplastics over a 30-day period.

The following equation was used to calculate the estimated tissue dose (in µGy). Equivalent dose was calculated by multiplying the absorbed dose to the organ or tissue (DT) with the radiation weighting factor, W_R_ [56]:

(4) $$\mathrm{Tissue}\;\mathrm{Dose}(\mu \mathrm{Gy})=\left(\frac{C_{\mathrm{ads}}}{1000}\times IR\times t\right)\times CF\times w_{R}$$

where:

- $C_{\mathrm{ads}}$ is the adsorption concentration of the radionuclide on microplastics (mBq/kg);
- IR is the ingestion rate of microplastics (0.5 g/day);
- t is the exposure duration (30 days);
- CF is the dose conversion factor (0.001 µGy/mBq);
- $w_{R}$ is the radiation weighting factor, taken as 20 for Ra-226 (alpha emitter) and 1 for Cs-137 and K-40 (beta/gamma emitters).

The results are presented in Table 3. Ra-226 yielded the highest estimated dose across all sampling points, particularly at point 1 (0.004 µGy), which was closest to the nuclear power plant. Cs-137 and K-40 contributed significantly lower doses, with maximum estimated tissue doses of 0.0001 µGy and 0.00009 µGy, respectively.

These values suggest that among the radionuclides studied, Ra-226 poses the greatest potential toxicological risk due to its high adsorption capacity and alpha radiation, which has greater biological effectiveness. Cs-137, while present in lower concentrations, is still of concern due to its potential to cause oxidative stress and reproductive toxicity. K-40, a naturally occurring radionuclide, showed the weakest adsorption and the lowest dose contribution.

Although the calculated tissue doses are relatively low, it is important to consider the cumulative effects of chronic exposure, bioaccumulation, and trophic transfer. Ingested radionuclide-laden microplastics may localize in the gastrointestinal tracts of marine organisms, leading to sustained internal exposure. Alpha radiation from Ra-226, even at low doses, is particularly damaging at the cellular level, potentially causing DNA strand breaks, chromosomal aberrations, and impaired cell function. These findings underscore the necessity of including toxicological endpoints in future studies, such as oxidative stress biomarkers, DNA damage assays, and histopathological evaluations, to validate and refine the predicted risk associated with compound microplastic–radionuclide pollution in marine environments.

## 4. Conclusions

The accumulation of microplastics in the marine environment has become one of the most significant global environmental pollution issues. Due to their long persistence and slow degradation, microplastics not only threaten ecosystems but also adsorb various harmful substances, including heavy metals, organic pollutants, and radionuclides. Research has shown that the adsorption of radionuclides by microplastics varies significantly under different environmental conditions, particularly influenced by particle size and environmental depth, with distinct trends in adsorption capacity.

The experimental data from this study indicate that the adsorption capacity of microplastics for different radionuclides varies significantly. Ra-226 and Cs-137 exhibited higher adsorption concentrations, particularly on microplastics mixed with the bottom water. The concentration on microplastics mixed with the bottom water of Ra-226 was 13.29 mBq/kg, while the concentration on microplastics mixed with the surface water was 1.65 mBq/kg. The Cs-137 concentration on microplastics mixed with the bottom water was 6.99 mBq/kg, compared to 1.31 mBq/kg on microplastics mixed with the surface water, showing strong adsorption of these radionuclides by microplastics. In contrast, K-40, as a naturally occurring radionuclide, exhibited a weaker adsorption capacity on microplastics, with a concentration on microplastics mixed with the bottom water of 2.1 mBq/kg and a concentration on microplastics mixed with the surface water of 0.35 mBq/kg.

The impact of microplastic particle size on adsorption capacity was also clearly demonstrated in this study. As the particle size of microplastics increased, their adsorption capacity significantly decreased. Microplastics with a 50 µm particle size showed adsorption concentrations of 13.29 mBq/kg for Ra-226 and 6.99 mBq/kg for Cs-137, while the concentrations for 100 µm and 150 µm particles decreased to 3.14 mBq/kg and 1.39 mBq/kg, and 2.2 mBq/kg and 0.35 mBq/kg, respectively. This indicates that smaller-sized microplastics, due to their larger surface area, are more efficient in adsorbing radionuclides and could play a greater role in the transport of pollution in the environment.

In conclusion, the presence of microplastics in the environment is not just a form of pollution but also serves as a carrier of other pollutants, especially radionuclides. The binding mechanisms between microplastics and radionuclides are closely related to surface properties, particle size, and environmental conditions. The adsorption capacity of microplastics for radionuclides directly influences the spread and accumulation of these pollutants, potentially posing serious threats to marine life, ecosystems, and human health through bioaccumulation in the food chain. Future research should focus on long-term field studies to assess the real-world behavior of radionuclide-contaminated microplastics, including their transport, degradation, and bioaccumulation. Additionally, further investigation is needed into how different types of polymers affect radionuclide adsorption capacity, as well as the combined impact of environmental factors such as pH, temperature, salinity, organic matter, and the surface area changes in aged microplastics on adsorption efficiency. These insights will help improve pollution management strategies and mitigate potential risks to ecosystems and human health.

## Figures and Tables

**Table 1:** Sampling site coordinates, water depths, and collection details (surface samples were taken at ~20 cm depth using a depth-specific glass water sampler; each sampling lasted approximately 5 min).

Sampling Point	Coordinates (E, N)	Surface Sample Depth (m)	Bottom Sample Depth (m)	Water Volume per Layer	Approx. Sampling Time (hh:mm)
Point 1	119°29.826′ E, 34°41.339′ N	0.2	4	10 L	~08:03
Point 2	119°34.271′ E, 34°45.582′ N	0.2	6	10 L	~08:35
Point 3	119°41.776′ E, 34°53.104′ N	0.2	13	10 L	~09:12
Point 4	119°47.438′ E, 34°59.264′ N	0.2	23	10 L	~09:53

**Table 2:** Based on water samples collected from four different sampling sites around Tianwan Nuclear Power Plant (as shown in Table 1), PVC microplastics of various sizes (in μm) were added. The initial concentrations (*C*_0_) and equilibrium concentrations (*C_e_*) of radionuclides (Ra-226, Cs-137, and K-40) adsorbed on the microplastics were analyzed.

Radionuclides	Water Sample Point	*C*_0_ (mBq/L)	MPs(μm) Size	*C_e_* (mBq/L)
Ra-226	point 1	25.72	50	21.8
	point 1	25.72	100	23.5
	point 1	25.72	150	24.3 ± 1.2
	point 2	9.51	50	8.1 ± 0.4
	point 2	9.51	100	8.9 ± 0.4
	point 2	9.51	150	9.2 ± 0.5
	point 3	6.14	50	4.9 ± 0.2
	point 3	6.14	100	5.5 ± 0.3
	point 3	6.14	150	5.8 ± 0.3
	point 4	2.89	50	2.1 ± 0.1
	point 4	2.89	100	2.6 ± 0.1
	point 4	2.89	150	2.8 ± 0.1
Cs-137	point 1	11.53	50	9.2 ± 0.5
	point 1	11.53	100	10.1 ± 0.5
	point 1	11.53	150	10.7 ± 0.5
	point 2	5.26	50	4.3 ± 0.2
	point 2	5.26	100	4.8 ± 0.2
	point 2	5.26	150	5.1 ± 0.3
	point 3	2.59	50	1.9 ± 0.1
	point 3	2.59	100	2.2 ± 0.1
	point 3	2.59	150	2.4 ± 0.1
	point 4	1.35	50	0.9 ± 0.05
	point 4	1.35	100	1.1 ± 0.05
	point 4	1.35	150	1.3 ± 0.1
K-40	point 1	6.19	50	5.7 ± 0.3
	point 1	6.19	100	5.9 ± 0.3
	point 1	6.19	150	6.0 ± 0.3
	point 2	3.11	50	2.8 ± 0.1
	point 2	3.11	100	2.9 ± 0.1
	point 2	3.11	150	3.0 ± 0.1
	point 3	1.28	50	1.1 ± 0.1
	point 3	1.28	100	1.2 ± 0.1
	point 3	1.28	150	1.3 ± 0.1
	point 4	0.56	50	0.4 ± 0.02
	point 4	0.56	100	0.5 ± 0.03
	point 4	0.56	150	0.6 ± 0.03

**Table 3:** Estimated intake and tissue dose from 50 µm microplastic-bound radionuclides.

Radionuclide	Sampling Point	Adsorption (mBq/kg)	Estimated Intake (mBq)	Estimated Tissue Dose (µGy)
Ra-226	Point 1	13.29	0.19935	0.00399
Ra-226	Point 2	8.10	0.12150	0.00243
Ra-226	Point 3	4.90	0.07350	0.00147
Ra-226	Point 4	2.10	0.03150	0.00063
Cs-137	Point 1	6.99	0.10485	0.00010
Cs-137	Point 2	4.30	0.06450	0.00006
Cs-137	Point 3	1.90	0.02850	0.00003
Cs-137	Point 4	0.90	0.01350	0.00001
K-40	Point 1	5.70	0.08550	0.00009
K-40	Point 2	2.80	0.04200	0.00004
K-40	Point 3	1.10	0.01650	0.00002
K-40	Point 4	0.40	0.00600	0.00001

## Data Availability

The original contributions presented in this study are included in the article material. Further inquiries can be directed to the corresponding authors.
